# How Does Nutritional Status Affect Outcomes in Patients with Neurological Diseases?

**DOI:** 10.18502/ijph.v49i10.4689

**Published:** 2020-10

**Authors:** Şükran GÜZEL, Eda GÜRÇAY, Ebru KARACA UMAY, Serdar MERCİMEKÇİ, Aytül ÇAKCI

**Affiliations:** Physical Medicine and Rehabilitation Clinic, Ankara Diskapi Yildirim Beyazit Education and Research Hospital, University of Health Sciences, Ankara, Turkey

**Keywords:** Stroke, Malnutrition, Function, Rehabilitation

## Abstract

**Background:**

To evaluate the nutritional status of patients with neurological diseases during the rehabilitation process and to investigate the relationships between the nutritional status and disease severity and clinical evaluation outcomes.

**Methods:**

In this prospective trial, 109 patients with a disease duration of <6 months, hospitalized for neurological rehabilitation in Physical Medicine and Rehabilitation Clinic, Ankara, Turkey were enrolled from 2014–17. All patients were assessed with the Mini Mental State Examination (MMSE) test, European Quality of Life Scale (Euro-QoL), Hospital Anxiety and Depression Scale (HADS), Pittsburg Rehabilitation Participation Scale (PRPS), and Functional Ambulation Category (FAC). Nutritional status was analyzed by biochemical and anthropometric parameters. The patients received a conventional rehabilitation program and a nutritional support according to clinical and laboratory findings for 4 weeks. The outcome data were evaluated at baseline and at the end of 4-week treatment.

**Results:**

Linear regressions analysis revealed that the significant independent predictors that associated positively with baseline insulin (*P*=0.010) and negatively with baseline cortisol (*P*=0.020) levels were Brunnstrom upper and hand stages. Additionally, the significant independent predictor that associated positively with baseline insulin (*P*=0.041) was Brunnstrom lower stage.

**Conclusion:**

Insulin and cortisol levels may be predictors in motor function recovery of stroke patients in rehabilitation process. Early detection and treatment of malnutrition both during hospitalization and follow-up might be important for the improvement of outcomes.

## Introduction

Malnutrition is nutritional deficiency or excess, caused by the imbalance between the needs of the body and the amount taken. Malnutrition significantly affects both physical and cognitive functions, resulting in increased morbidity, mortality and direct/indirect cost of society (1).

Neurological and neurosurgical diseases differ according to lesion, injury, life expectancy, residual mobility-functionality, and drug treatment. On the other hand, diseases with different physiopathology, location, and evolution can be presented with similar clinical pictures (2). Neurological diseases are frequently associated with malnutrition. The causes of malnutrition include oropharyngeal dysphagia, unconsciousness, perception deficits, cognitive dysfunctions, and increased needs. Malnutrition negatively affects patients’ rehabilitation process and functional recovery (3–5).

Specific proteins and other biochemical markers are indicators used to determine nutritional status. The most important markers are albumin, transferrin, pre-albumin and physical measurements of nitrogen and creatinine/height index (6). Anthropometric data such as body mass index (BMI), ideal body weight, triceps skin fold thickness (TSF), middle upper arm circumference (MUAC) and calf circumference (CC) are widely used tools for nutritional assessment.

Protocols developed to detect malnutrition in adults are based on changes in the acute phase proteins (7, 8). Although these laboratory tests are indicative of possible inflammation, they do not specifically indicate malnutrition and typically do not respond to nutritional interventions in adjusting the active inflammatory response (9). Because of its lower half-life and the smaller size as a constituent, the transferrin is a better indicator of nutritional status than albumin. The other biochemical indicator is prealbumin with a very short half-life, an excellent nutritional index and a marker of response to nutritional therapy (10). Total lymphocyte count is an indicator of immune function that correlates with albumin and decreases during food consumption. Serum hemoglobin and hematocrit may reflect a general state of malnutrition (11).

Although there are many studies on malnutrition, to our knowledge, there is no study of which tests show malnutrition and whether nutrition parameters affect rehabilitation results in patients with neurological disease.

Therefore, the aims of this study were to evaluate the nutritional status of patients with neurological diseases who referred to the rehabilitation clinic and to investigate the relationships between the nutritional status and disease severity and clinical evaluation outcomes.

## Materials and Methods

### Study Design and Patients

One hundred and nine patients with a disease duration of <6 months, admitted to Physical Medicine and Rehabilitation Clinic, Ankara, Turkey for neurological rehabilitation between Jan 2014 and Jan 2017 were included in this study. Patients between the ages of 18–80 yr, who had no nutritional assessment for the last 6 months, had not received any food supplements, and/or had no interruption in diet for 5 d and had no severe metabolic/endocrine disease were included in this study.

The protocol was explained to all participants, and informed consent was obtained at the beginning of the study. The ethics committee of the Institute approved the study protocol, and all procedures were performed in compliance with the Helsinki Declaration.

Demographic and clinical data including age, gender, educational level, comorbidity, smoking and alcohol status, etiology, disease duration and severity were recorded.

### Clinical Evaluation Outcomes

Brunnstrom motor stages for stroke patients, American Spinal Injury Association scale (ASIA) for spinal cord injury (SCI) patients and the Disability Rating Scale (DRS) for traumatic brain injury (TBI) patients were used.

All patients were assessed with the Mini Mental State Examination (MMSE) test, European Quality of Life Scale (Euro-QoL), Hospital Anxiety and Depression Scale (HADS), Pittsburg Rehabilitation Participation Scale (PRPS), and Functional Ambulation Category (FAC).

MMSE test score was used to evaluate cognitive functions as maximum score 30 and a score equal to or greater than 24 were considered normal (12).

Euro-QoL is an instrument which evaluates the generic quality of life. The Euro-QoL descriptive system is a measure with one question for each of the five dimensions that include mobility, self-care, usual activities, pain/discomfort, and anxiety/depression. The questionnaire includes a visual analog scale, by which respondents can report their perceived current health status with a grade ranging from 0 (the worst possible health status) to 100 (the best possible health status) (13).

The HADS aims to measure symptoms of anxiety and depression and consists of 14 items, seven items for the anxiety subscale (HADS-A) and seven for the depression subscale (HADS-D). Each item is scored on a response-scale with four alternatives ranging between 0 and 3 (14). Pittsburgh Rehabilitation Participation Scale (PRPS), a clinician-rated 6-point Likert-type item, measuring patient participation (15). The evaluation of ambulation with FAC is based on 6 scores between 0–5 (16).

### Nutritional Status Evaluation Parameters

Malnutrition was assessed by BMI, serum albumin, prealbumin, total lymphocyte count, transferrin, hemoglobin and hematocrit levels. Biochemically, erythrocyte sedimentation rate (ESR), C reactive protein (CRP), hemoglobin, iron, total iron binding capacity (TIBC), total cholesterol, low density lipoprotein (LDL), high density lipoprotein (HDL), triglyceride, thyroid function tests (Thyrotropin-Stimulating Hormone-TSH, T3, T4), insulin, cortisol, zinc, magnesium, B12 and D vitamins were evaluated.

All anthropometric measurements, including triceps skin-fold thickness (TSF), mid-upper arm circumference (MUAC), and calf circumference (CC) were performed by a single observer. The CC was measured from the widest part of the calf while the patient was in the sitting position (17). MUAC was measured with a tape measurer from the midpoint of the triceps on the dominant or non-paretic arm between the acromion and the olecranon processes. TSF was measured at the midpoint with a skinfold caliper to the nearest 0.2 cm.

### Therapy Protocol

All patients received a conventional rehabilitation program, 5 d a week lasting 30 min each, for 4 wk including range of motion, stretching and strengthening exercises, for 4 weeks. In addition, a nutritional support was established according to biochemical and anthropometric parameters of subjects and applied for 4 weeks. The outcomes were evaluated at baseline and at 4 weeks.

### Statistical Analysis

SPSS 25.0 for Windows software (Chicago, IL, USA) was used in the analysis of the data. In descriptive statistics, the data were expressed as mean±standard deviation (SD) for continuous variables, and as frequencies and percentages (%) for nominal variables. The Kolmogorov-Smirnov test was used to determine whether continuous variables had a normal distribution. The Wilcox-on Sign test was used to detect statistical significance between recurrent measurements. Bonferroni correction was applied to control possible Type I errors in comparison. Pearson correlation test was used to establish the relationship between the changes of outcome parameters and the baseline nutritional measures as well as linear regression analysis was performed for significant correlations. Statistical significance level was set at *P*<0.05.

## Results

Mean disease duration among stroke patients was 2.21 (SD 1.15) months, SCI patients was 2.18 (SD 2.01) months, and TBI patients was 3.48 (SD 2.11) months. Demographic and clinical characteristics of the patients are presented in Table 1. Malnutrition was found in 51 patients (46.8%) before therapy and 30 patients (27.5%) after 4 weeks.

**Table 1:** Demographic and clinical characteristics of the patients

***Variable***	***Mean (SD), n (%) n=109***
Age (yr)	50.94 (20.74)
Gender	
Female	67 (61.5)
Male	42 (38.5)
Education level	
Illerated	13 (11.9)
<5 yr	36 (33.0)
5 yr	16 (14.7)
8 yr	22 (20.2)
11 yr	22 (20.2)
Comorbidity	
DM	28 (28.7)
HT	35 (32.1)
Hypothyroidism	3 (2.7)
Heart disease	8 (7.39
Asthma	1 (0.9 )
Smoking status	
Presence	22 (20.2)
Absence	87 (79.8)
Alcohol status	
Presence	7 (6.4)
Absence	102 (93.6)
Etiology	
SCI	28 (25.7)
Stroke	54 (49.5)
TBI	27 (24.8)
Disease severity	
ASIA (SCI)	
A	16 (57.2)
B	6 (21.4)
C	4 (14.3)
D	2 (7.1)
Brunnstrom stage (Stroke)	
Hand	2.48 (1.92)
Upper extremity	2.44 (1.86)
Lower extremity	3.40 (1.58)
DRS (TBI)	19.56 (5.50)

SD, standard deviation; DM, Diabetes mellitus; HT, Hypertension; SCI, Spinal cord injury; TBI, Traumatic brain injury; ASIA, American spinal injury association; DRS, Disability rating scale

After therapy, Brunnstrom stage was 2.80 (SD 2.09) for upper extremity, 2.70 (SD 2.11) for hand, and 3.48 (SD 1.41) for lower extremity. DRS was found to be 12.63 (SD 7.99). The changes in Brunnstrom stages for hand (*P*=0.011) and upper extremity (*P*=0.017) and DRS (*P*=0.001) were all significant. On the other hand there was no ASIA change. Significant improvement was observed for MMSE, Euro-QoL, HADS-D, PRPS, and FAC (Table 2).

**Table 2:** Comparisons of clinical evaluation parameters before and after therapy

***Variable***	***Before therapy Mean (SD)***	***After therapy Mean (SD)***	***P***
MMSE	20.43 (12.64)	24.71 (9.13)	.001
Euro-QoL	12.33 (1.79)	11.08 (2.50)	.001
HADS-A	5.38 (3.88)	5.07 (3.48)	.036
HADS-D	4.16 (4.90)	4.28 (4.97)	.001
PRPS	3.55 (1.68)	4.04 (1.56)	.001
FAC	1.44 (0.78)	2.33 (1.01)	.001

SD, Standard deviation; MMSE, Mini Mental State Examination; Euro-QoL, European Quality of life Scale; HADS, Hospital anxiety and depression scale; PRPS, Pittsburg Rehabilitation Participation Scale; FAC, Functional ambulation category.

A value of *P*<0.025 was considered statistically significant

While the patients with oral feeding were 85.3% (n=93) during the admission, this rate increased to 91.7% (n=100) at discharge.

Statistically significant changes were detected biochemically in lymphocyte count, total protein, ESR, CRP, iron, total cholesterol, HDL, LDL, insulin, Zn, B12 and D vitamin levels. In the anthropometric measurements, significant changes were observed in TSF, MUAC, CC and BMI (Table 3). Correlation analysis between baseline nutritional status and disease severity and clinical evaluation outcomes are shown in Tables 4 and 5.

**Table 3:** Comparisons of nutritional evaluation parameters before and after therapy

***Parameters***	***Before therapy Mean (SD), n (%)***	***After therapy Mean (SD), n (%)***	***P***
Lymphocyte (900–2900 /microliters)	1551.39 (420.31)	1905.0 (511.41)	0.001
Albumin (3.5–5.2 g/dl)	3.55 (0.55)	3.85 (0.46)	0.124
Prealbumin (20–40 mg/dl)	20.27 (7.14)	21.85 (7.33)	0.271
ESR (<20 mm/h)	42.51 (29.53)	31.39 (25.59)	0.005
CRP (<3 mg/dl)	55.87 (58.50)	25.04 (27.47)	0.001
Iron (70–180 mcg/dl)	49.83 (40.06)	57.25 (31.57)	0.011
Iron bindıng capacity (155–355 mcg/dl)	190.32 (50.33)	213.41 (53.53)	0.035
Transferrin (215–380 mg/dl)	147.97 (53.62)	153.88 (65.93)	0.027
Total protein (6.6–8.3 g/dl)	5.98 (0.84)	6.46 (0.63)	0.024
Hemoglobin (13.0–17.3 g/dl)	10.52 (1.78)	12.24 (1.69)	0.027
Total cholesterol (0–200 mg/dl)	164.41 (50.15)	220.44 (34.94)	0.006
HDL (40–60 mg/dl)	30.81 (12.77)	46.72 (10.49)	0.011
LDL (<130 mg/dl)	102.55 (40.82)	114.65 (32.18)	0.019
Triglyceride (35–150 mg/dl)	119.86 (11.39)	129.41 (27.43)	0.028
T3 (2.5–3.9 pg/dl)	2.68 (0.54)	3.01 (1.09)	0.103
T4 (6.1–11.2 pg/dl)	6.46 (0.26)	7.40 (0.89)	0.127
TSH (0.34–5.6 mU/L)	1.84 (1.28)	1.74 (0.99)	0.393
Insulin (2.6–24.9 microunite/ml)	7.87 (16.71)	10.53 (11.59)	0.003
Cortisol (5–23 mcg/dl)	17.75 (6.63)	17.47 (11.06)	0.095
Zn (50–120 microgram/dl )	74.33 (16.36)	91.72 (16.35)	0.024
Mg (1.8–2.6 mg/dl)	1.95 (0.26)	1.91 (0.20)	0.213
Vitamin B12 (250–1100 pg/dl)	360.02 (237.40)	407.46 (310.46)	0.008
Vitamin D (20–30 ng/ml)	16.73 (9.95)	24.32 (11.92)	0.013
Triceps skin-fold thickness	9.02 (6.91)	15.46 (5.96)	0.001
Mid-upper arm muscle circumference	20.15 (4.63)	25.25 (4.50)	0.023
Calf circumference	20.06 (4.64)	28.64 (4.28)	0.018
BMI (kg/m^2^)	19.01 (5.17)	21.08 (4.84)	0.027

SD, Standard Deviation; ESR, erythrocyte sedimentation rate; CRP, C reactive Protein; HDL, high density lipoprotein; LDL, Low Density Lipoprotein; TSH, Thyrotropin-Stimulating Hormone; Zn, Zinc; Mg, Magnesium; BMI, Body mass index.

A value of *P*<0.025 was considered statistically significant

**Table 4:** Correlation analysis between nutritional status and disease severity outcomes

***Parameters***	***Br-upper r*/P**	***Br-hand r*/P**	***Br-lower r*/P**	***ASIA r*/P**	***DRS r*/P**
Lymphocyte (900–2900 /microliters)	0.357/0.014	0.408/0.004	0.506/0.001	0.185/0.423	0.039/0.853
Albumin (3.5–5.2 g/dl)	0.192/0.201	0.204/0.174	0.299/0.053	0.332/0.141	0.073/0.730
Prealbumin (20–40 mg/dl)	0.086/0.566	0.082/0.584	0.096/0.522	0.262/0.264	0.024/0.911
ESR (<20 mm/h)	−0.173/0.590	−0.173/0.590	−0.124/0.701	−0.578/0.052	−0.228/0.321
CRP (<3 mg/dl)	−0.189/0.557	−0.189/0.502	−0.183/0.569	−0.348/0.203	−0.090/0.698
Iron (70–180 mcg/dl)	−0.121/0.709	−0.121/0.709	−0.090/0.780	−0.711/0.053	−0.125/0.591
Iron bindıng capacity (155–355 mcg/dl)	0.267/0.402	0.262/0.407	0.308/0.330	0.480/0.070	0.262/0.251
Transferrin (215–380 mg/dl)	0.191/0.203	0.283/0.057	0.239/0.109	0.002/0.995	0.003/0.623
Total protein (6.6–8.3 g/dl)	−0.104/0.461	−0.104/0.761	−0.093/0.985	−0.259/0.332	−0.021/0.938
Hemoglobin (13.0–17.3 g/dl)	0.233/0.467	0.233/0.467	0.081/0.803	0.700/0.051	0.256/0.263
Total cholesterol (0–200 mg/dl)	−0.479/0.115	−0.489/0.115	−0.592/0.042	−0.266/0.319	−0.119/0.607
HDL (40–60 mg/dl)	0.261/0.412	0.261/0.412	0.295/0.353	0.358/0.174	0.062/0.891
LDL (<130 mg/dl)	−0.479/0.115	−0.479/0.115	−0.589/0.044	0.369/0.160	0.113/0.626
Triglyceride (35–150 mg/dl)	−0.330/0.265	−0.330/0.265	−0.388/0.212	−0.353/0.180	−0.069/0.767
T3 (2.5–3.9 pg/dl)	−0.088/0.785	−0.088/0.785	−0.161/787	−0.093/0.732	−0.016/0.946
T4 (6.1–11.2 pg/dl)	−0.432/0.161	−0.432/0.161	−0.334/0.789	−0.002/0.995	−0.147/0.594
TSH (0.34–5.6 mU/L)	−0.567/0.055	−0.567/0.055	−0.421/0.123	−0.005/0.955	−0.146/0.529
Insulin (2.6–24.9 microunite/ml)	0.655/0.021	0.655/0.021	0.608/0.036	0.146/0.604	0.265/0.245
Cortisol (5–23 mcg/dl)	−0.580/0.048	−0.580/0.048	−0.384/0.218	−0.004/0.989	−0.113/0.627
Zn (50–120 microgram/dl)	−0.393/0.207	−0.393/0.207	−0.504/0.095	−0.080/0.778	−0.246/0.283
Mg (1.8–2.6 mg/dl)	−0.407/0.190	−0.407/0.190	−0.589/0.051	−0.470/0.066	−0.289/0.205
Vitamin B12 (250–1100 pg/dl)	0.042/0.896	0.042/0.896	0.108/0.832	0.203/0.450	0.040/0.862
Vitamin D (20–30 ng/ml)	0.079/0.807	0.079/0.807	0.166/0.605	0.229/0.393	0.175/0.449
Triceps skin-fold thickness	0.024/0.942	0.024/0.942	0.107/0.741	0.379/0.148	0.293/0.197
Mid-upper arm muscle circumference	0.084/0.644	0.202/0.258	0.021/0.908	0.110/0.636	0.209/0.316
Calf circumference	0.055/0.760	0.007/0.970	0.087/0.629	0.208/0.378	0.189/0.366
BMI (kg/m^2^)	0.010/0.948	0.091/0.544	0.143/0.338	0.259/0.258	0.085/0.634

r, correlation coefficient; Br, Brunnstrom; ASIA, American Spinal Injury Association; DRS, Disability rating scale; ESR, erythrocyte sedimentation rate; CRP, C reactive Protein; HDL, High density lipoprotein; LDL, Low Density Lipoprotein; TSH, Thyrotropin-Stimulating Hormone; Zn, Zinc; Mg, Magnesium; BMI, Body mass index.

A value of *P*<0.05 was considered statistically significant

**Table 5:** Correlation analysis between nutritional status and clinical evaluation outcomes

***Parameters***	***MMSE r*/P**	***FAC r*/P**	***Euro-QoL r*/P**	***HADS-D r*/P**	***HADS-A r*/P**	***PRPS r*/P**
Lymphocyte (900–2900 /microliters)	0.024/0.885	0.144/0.300	−0.223/0.105	−0.138/0.145	−0.104/0.565	−0.031/0.825
Albumin (3.5–5.2 g/dl)	0.177/0.280	0.258/0.062	−0.055/0.986	−0.138/0.348	−0.044/0.810	−0.172/0.217
Prealbumin (20–40 mg/dl)	−0.192/0.242	−0.072/0.696	−0.031/0.827	−0.130/0.380	−0.131/0.475	−0.056/0.638
ESR (<20 mm/h)	−0.139/0.393	−0.129/0.352	−0.146/0.445	0.087/0.552	0.071/0.693	0.234/0.089
CRP (<3 mg/dl)	0.091/0.581	−0.051/0.785	0.165/0.287	0.164/0.237	0.069/0.706	0.039/0.780
Iron (70–180 mcg/dl)	−0.134/0.416	−0.054/0.699	0.013/0.925	−0.019/0.896	−0.058/0.754	0.044/0.746
Iron bındıng capacity (155–355 mcg/dl)	0.101/0.512	0.250/0.871	−0.291/0.055	0.045/0.761	−0.083/0.650	0.160/0.253
Transferrin (215–380 mg/dl)	0.262/0.107	0.201/0.149	−0.236/0.089	0.244/0.095	0.023/0.900	0.221/0.112
Total protein (6.6–8.3 g/dl)	−0.269/0.098	0.246/0.076	−0.020/0.885	−0.156/0.290	−0.072/0.696	−0.193/0.167
Hemoglobin (13.0–17.3 g/dl)	−0.098/0.589	0.218/0.114	−0.195/0.157	0.098/0.502	0.086/0.633	0.108/0.435
Total cholesterol (0–200 mg/dl)	−0.034/0.835	−0.042/0.763	−0.070/0.613	−0.066/0.563	−0.051/0.766	0.123/0.376
HDL (40–60 mg/dl)	0.164/0.313	0.211/0.125	−0.212/0.124	−0.016/0.916	0.099/0.584	0.162/0.243
LDL (<130 mg/dl)	−0.087/0.592	−0.012/0.929	−0.074/0.597	−0.023/0.874	−0.057/0.851	0.152/0.271
Triglyceride (35–150 mg/dl)	−0.012/0.939	−0.250/0.069	0.220/0.110	−0.067/0.649	−0.043/0.812	−0.024/0.865
T3 (2.5–3.9 pg/dl)	−0.075/0.647	−0.007/0.958	−0.097/0.488	−0.087/0.551	−0.183/0.507	−0.030/0.837
T4 (6.1–11.2 pg/dl)	−0.071/0.665	−0.015/0.916	0.109/0.433	−0.019/0.895	−0.084/0.641	−0.122/0.380
TSH (0.34–5.6 mU/L)	−0.222/0.168	−0.257/0.061	0.193/0.163	−0.102/0.484	−0.001/0.997	−0.119/0.393
Insulin (2.6–24.9 micro-unite/ml)	0.179/0.275	−0.157/0.362	−0.023/0.868	−0.081/0.584	−0.119/0.556	−0.069/0.621
Cortisol (5–23 mcg/dl)	−0.187/0.255	0.043/0.758	−0.055/0.697	0.026/0.860	−0.119/0.517	−0.088/0.529
Zn (50–120 microgram/dl )	−0.108/0.514	−0.197/0.157	−0.069/0.725	−0.134/0.363	0.049/0.792	−0.251/0.069
Mg (1.8–2.6 mg/dl)	−0.125/0.442	0.174/0.209	−0.123/0.376	−0.011/0.942	−0.001/0.976	0.030/0.832
Vitamin B12 (250–1100 pg/dl)	0.001/0.998	0.098/0.432	0.178/0.198	0.158/0.279	0.205/0.253	−0.006/0.966
Vitamin D (20–30 ng/ml)	−0.052/0.748	0.148/0.284	−0.091/0.514	0.148/0.310	0.289/0.103	−0.075/0.591
Triceps skin-fold thickness	0.103/0.598	0.321/0.052	0.119/0.389	−0.073/0.617	−0.007/0.970	0.097/0.403
Mid-upper arm muscle circumference	0.172/0.288	0.159/0.251	−0.113/0.415	0.016/0.912	−0.003/0.986	0.162/0.142
Calf circumference	−0.354/0.059	0.114/0.102	−0.045/0.452	0.035/0.814	0.0190.938	0.231/0.100
BMI (kg/m^2^)	0.238/0.140	−0.176/0.203	−0.032/0.808	−0.029/0.846	0.060/0.739	0.198/0.152

r, correlation coefficient; MMSE, Mini Mental State Examination; FAC, Functional ambulation categories; Euro-QoL, European Quality of life Scale; HADS, Hospital anxiety and depression scale; ESR, erythrocyte sedimentation rate; CRP, C reactive Protein; HDL, high density lipoprotein; LDL, Low Density Lipoprotein; TSH, Thyrotropin-Stimulating Hormone; Zn:, Zinc; Mg, Magnesium; BMI, Body mass index.

A value of *P*<0.05 was considered statistically significant

On multiple linear regressions analysis, the significant independent predictors that associated positively with baseline insulin (*P*=0.010) and negatively with baseline cortisol (*P*=0.020) levels were Brunnstrom upper and hand stages. Additionally, the significant independent predictor that associated positively with baseline insulin *(P*=0.041) was Brunnstrom lower stage.

## Discussion

In this study, nutritional status of patients with neurological diseases were evaluated in terms of BMI, biochemical parameters and anthropometric measurements. All patients received a conventional rehabilitation program and a nutritional support according to clinical and laboratory outcomes. Insulin and cortisol levels were found to be effective factors for motor function recovery level.

Nutritional disorders and malnutrition are common in neurological diseases. In these diseases, food intake reduced depending on many different reasons such as swallowing problems. The catastrophic process developing during acute period greatly increases the energy and protein requirement. In the chronic period, disturbances in nutrient uptake reaches important dimensions depending on factors such as functional disorders (paresis, ataxia, apraxia, involuntary movements, visual defects etc.), emotional changes (depression, anxiety etc.), autonomic disorders (anorexia, nausea, vomiting, constipation, delayed gastric emptying, reflux etc.), cognitive impairment, dysphagia, and side effects related to medications used.

As a result, there is a decrease in muscle and bone mass, decrease in mobility, decrease in quality of life, prolonged stay in hospital, increase in treatment-care costs, increase in complications such as infection, delay in pressure ulcers healing and increase in mortality (18). As a result, there may be decrease in muscle and bone mass, mobility, and quality of life, prolongation of hospital stay, delay in healing ulcers, increase in treatment-care costs, complications such as infection, and mortality.

Ninety-eight patients with subacute brain injury were evaluated in terms of nutrition with a screen test, 30% of patients were at high risk for malnutrition, and had weight loss and 52% of patients received enteral or parenteral nutrition at admission. The rehabilitation period with nutritional support has resulted in weight gain. Furthermore, this occurs a positive effect on rehabilitation outcomes (19). However, in this study, there is no relationship between risk of malnutrition and severity of injury, complications, functional outcome or duration of stay.

Kaur et al. assessed nutritional status of adults in ambulatory rehabilitation with bioelectrical impedance. Functional performance did not show a difference for participants assessed as at risk of malnutrition or malnourished compared to the well-nourished, but the SF-36 mental component score was significantly higher for those who were well nourished (20). Overall, 483 patients were evaluated with acute stroke malnourished during admission. They assessed malnutrition with BMI, serum albumin and total cholesterol levels. Subjects with poor functional outcomes were found to have malnourished and had longer hospital stays. Malnutrition in acute stroke was an independent risk factor for poor functional outcomes (21). In other study involving 103 stroke patients, the nutritional (a screen test) and functional status (through the Barthel index and the modified Rankin scale) of the patients were assessed. Malnutrition and risk of malnutrition were found in 8.2% and 38.1% patients, respectively. In conclusion, nutritional deficiency was associated with poorer functioning and quality of life (22).

In the literature, there is no study showing the association between of insulin and cortisol levels on motor function recovery. Moreover, none of the above-mentioned studies have shown a direct effect of one of the parameters or screen tests. After brain or spinal cord injury occurs the impairment of autoregulation and glucose levels are increased. In some studies, dysregulated glucose metabolism has also been shown to be prolonged for months after stroke (23). A neuroendocrine stress response and an inflammatory response may also play a role in generating hyperglycemia (24). The cortisol, one of the core regulators of both the stress response and plasma glucose concentration, is directly neurotoxic and inhibits recovery after brain injury. We included patients with neurological disorders in early period. Therefore, we may have found a relationship between motor function recovery and insulin and cortisol levels in stroke patients. We found no relationship between these parameter levels and the DRS and ASIA scale. This might be depend on that DRS is a more comprehensive scale that evaluates disability in all aspects, not only motor recovery but also cognitive abilities. Similarly, the ASIA scale includes both the motor and the sensory levels.

These results are important because it suggests that the catabolic process may continue in the rehabilitation period, even if BMI and albumin levels are normal. We consider that initial higher ESR and CRP levels and a lower total lymphocyte count together support this view. Insulin level depends on many parameters such as insulin resistances, a significant limitation of our study was the lack of serum glucose level evaluation which is unlikely to calculate insulin resistance. Another limitation of our study was that the duration of stay in critical care services associated with nutritional status was not noted.

## Conclusion

Insulin and cortisol levels may be predictors in motor function recovery of stroke patients in rehabilitation process. Strategies should be developed for early detection and treatment of malnutrition both during hospitalization and follow-up. Multidisciplinary team work and the implementation of specific nutritional interventions can enhance the improvement of outcomes in neurological diseases.

## Ethical considerations

Ethical issues (Including plagiarism, informed consent, misconduct, data fabrication and/or falsification, double publication and/or submission, redundancy, etc.) have been completely observed by the authors.
